# A comprehensive floristic knowledge of a fragment of Semideciduous Seasonal Forest [Parque Estadual da Serra da Concórdia], Rio de Janeiro, Brazil

**DOI:** 10.3897/BDJ.12.e125979

**Published:** 2024-10-04

**Authors:** Lara Serpa Jaegge Deccache, Claudine Massi Mynssen, Claudio Nicoletti de Fraga, Eduardo Pinheiro Fernandez, Elsie Franklin Guimarães, Elton John de Lírio, Fabiana Luiza Ranzato Filardi, Fernanda Ribeiro de Mello Fraga, Fernanda Saleme, Gustavo Hiroaki Shimizu, Haroldo Cavalcante de Lima, Helen Ayumi Ogasawara, Hemily Oliveira Marques, Isabela Maciel Waga, Isabella Cristina de Castro Silva, Jenifer de Carvalho Lopes, Leonardo Biral, Lucas Vieira Lima, Maria Liris Barbosa, Mario Gomes, Massimo Giuseppe Bovini, Miriam Kaehler, Nádia Roque, Otávio Luis Marques da Silva, Pedro Luís Rodrigues de Moraes, Rodrigo Lopes Borges, Ronaldo Marquete, Thuane Bochorny, Thiago Fernandes, Marcio Verdi

**Affiliations:** 1 Instituto de Pesquisas Jardim Botânico do Rio de Janeiro, Rio de Janeiro, Brazil Instituto de Pesquisas Jardim Botânico do Rio de Janeiro Rio de Janeiro Brazil; 2 Centro Nacional de Conservação da Flora, Rio de Janeiro, Brazil Centro Nacional de Conservação da Flora Rio de Janeiro Brazil; 3 IUCN SSC Brazil Plant Red List Authority, Rio de Janeiro, Brazil IUCN SSC Brazil Plant Red List Authority Rio de Janeiro Brazil; 4 Universidade de São Paulo, São Paulo, Brazil Universidade de São Paulo São Paulo Brazil; 5 Universidade Estadual de Campinas, Campinas, Brazil Universidade Estadual de Campinas Campinas Brazil; 6 Instituto Interamericano de Cooperação para Agricultura, Brasília, Brazil Instituto Interamericano de Cooperação para Agricultura Brasília Brazil; 7 Harvard University Herbaria, Cambridge, United States of America Harvard University Herbaria Cambridge United States of America; 8 Universidade Federal de Goiás, Goiânia, Brazil Universidade Federal de Goiás Goiânia Brazil; 9 Universidade Regional de Blumenau, Blumenau, Brazil Universidade Regional de Blumenau Blumenau Brazil; 10 Universidade Estadual de Feira de Santana, Feira de Santana, Brazil Universidade Estadual de Feira de Santana Feira de Santana Brazil; 11 Universidade Federal do Paraná, Curitiba, Brazil Universidade Federal do Paraná Curitiba Brazil; 12 Universidade Federal da Bahia, Salvador, Brazil Universidade Federal da Bahia Salvador Brazil; 13 Instituto de Pesquisas Ambientais do Estado de São Paulo, São Paulo, Brazil Instituto de Pesquisas Ambientais do Estado de São Paulo São Paulo Brazil; 14 Universidade Estadual Paulista Júlio de Mesquita Filho, Rio Claro, Brazil Universidade Estadual Paulista Júlio de Mesquita Filho Rio Claro Brazil; 15 Instituto Brasileiro de Geografia e Estatística, Rio de Janeiro, Brazil Instituto Brasileiro de Geografia e Estatística Rio de Janeiro Brazil

**Keywords:** Protected Areas, Semideciduous Seasonal Forest, Conservation, Catálogo de Plantas das Unidades de Conservação do Brasil

## Abstract

**Background:**

The "Serra da Concórdia" is part of the Atlantic Forest phytogeographical domain in the Brazilian state of Rio de Janeiro and it has a predominant phytophysiognomy of Semideciduous Seasonal Forest. This region underwent intense habitat loss and fragmentation during the 19^th^ century, due to coffee plantations and later pastures. With the decline of these activities, the areas were abandoned, triggering secondary succession. In 2002, the "Parque Estadual da Serra da Concórdia" was established in this region to preserve the remaining forest fragments. The updated list of vascular plants recorded in this protected area, published in the "Catálogo de Plantas das Unidades de Conservação do Brasil", is presented here, along with information on richness, endemism, and conservation status.

**New information:**

The "Parque Estadual da Serra da Concórdia" houses 231 vascular plant species, of which 90% are angiosperms, 10% ferns and lycophytes, and 27% endemic to the Atlantic Forest. Ten species are threatened with extinction, three are categorized as Endangered, and seven as Vulnerable. Although there have been expeditions in the "Parque Estadual da Serra da Concórdia", they have been limited, resulting in a low number of records and the species richness for a protected area. This is notable considering the 2,130 Brazilian native vascular plant species recorded in the semideciduous seasonal forest of Rio de Janeiro. Our data indicates that floristic inventories of Brazilian protected areas could help highlight gaps in flora knowledge and support the proposal of effective conservation actions.

## Introduction

The Atlantic Forest is one of 35 world’s hotspots of biodiversity (Myers et al. 2000, Mittermeier et al. 2011, Williams et al. 2011), areas that has undergone great loss of habitat and has a high level of endemism. The Atlantic Forest covers most of the coast of Brazil to northern Paraguay and Argentina, over a broad latitudinal (3°−30°S) and longitudinal (35°−60°W) range (Ribeiro et al. 2011). The heterogeneity of environments and landscapes divides its areas into different vegetation formations:

1. Dense Ombrophilous Forest,
2. Semideciduous Seasonal Forest,
3. High-altitude grassland,
4. Savanna-steppe,
5. Restinga, and
6. Mangrove (IBGE 2012).

Located fully within the Atlantic Forest phytogeographical domain, the state of Rio de Janeiro, in Southern-east Brazil, is a relevant area of occurrence for endemic and threatened plants (Coelho et al. 2017, Martinelli et al. 2018). Controversially, the Atlantic Forest has been historically undergoing rampant anthropic modification process since the 16^th^ century, with the European invasion of the South American continent (Martinelli et al. 2018, Maurenza et al. 2018). Despite this, 20% of its territory is covered by federal, state, and municipal protected areas (Maurenza et al. 2018).

The Semideciduous Seasonal Forest is among the most modified formations, affected by the historical processes of deforestation and timber exploitation in the Atlantic Forest (Fidalgo et al. 2009, Machado et al. 2021). Only 10% of its original area remains, consisting of forest fragments with little knowledge of its floristic characteristics and composition (Fidalgo et al. 2009, Machado et al. 2021).

The Serra da Concórdia is a mountain range located in an area covered with Seasonal Semideciduous Forest, inserted in the region of the hydrographic basin of the Rio Paraíba do Sul. The Serra da Concórdia has undergone intense fragmentation and deforestation mainly due to the introduction of coffee plantations and pasture in the 19^th^ century (Spolidoro 2001, Castro 2015, Coelho et al. 2017, Lazos-Ruíz et al. 2018). The growth of coffee production in the Paraíba do Sul valley was driven by the presence of native forests with soil rich in organic matter, which have been destroyed in this region (Lazos-Ruíz et al. 2018).

Later, a decline in coffee production took place, primarily due to the abolition of slavery and soil depletion, consequently pressing the inhabitants to leave the exploited sites (Castro 2015, Lazos-Ruíz et al. 2018). Then, the process of secondary succession began in some of the remaining vegetation fragments (Spolidoro 2001, Medeiros et al. 2020). As an example, around 63% of Serra da Concórdia is covered by forest, distributed in 72 fragments, of which 56 are smaller than 10 ha and vulnerable to biodiversity loss, and there are only two main fragments bigger than 1,000 ha (Caldas and Francelino 2009).

The "Parque Estadual da Serra da Concórdia" (PESC) comprises the two main forest fragments of the Serra da Concórdia (Caldas and Francelino 2009). The PESC was created in 2002 (Rio de Janeiro 2002) and was delimited by the forested area of the "Campo Experimental Santa Mônica", (formerly "Fazenda Santa Mônica") one of the pioneers in coffee production in the 19^th^ century. In 2016, it was expanded to include other areas in the "Serra da Concórdia" and "Serra de São Manuel" (Rio de Janeiro 2016, Maurenza et al. 2018).

The area of PESC was targeted for floristic studies conducted by Spolidoro (2001) prior to its official creation. The collection efforts dedicated to this protected area can be summarized in two main works: Spolidoro (2001), which recorded 85 species of flora in three plots within the "Campo Experimental Santa Mônica" area; and Maurenza et al. 2018, which compiled a list of 127 species for the PESC from databases. This was the first effort to compile a flora list of 36 state-protected areas of Rio de Janeiro, which significantly enhanced the understanding of the state's flora. It provided crucial data for protected area management plans and other territorial management documents, identified species of interest for research and conservation, and supplied informative material for environmental education activities.

The Catálogo de Plantas das Unidades de Conservação do Brasil (2023) presents lists of species occurring in different protected areas in Brazil. Apart from legal protection, these lists of species are fundamental as tools for decision-making and biodiversity conservation in the Brazilian phytogeographic domains (Maurenza et al. 2018). In addition, species richness and composition may indicate the need for collection efforts and scientific exploration of protected areas in Brazil. Here, we summarize the information from a recent inventory of the vascular flora of the PESC (Waga et al. 2023), published in the Catálogo de Plantas das Unidades de Conservação do Brasil (2023), and also provide details on richness, endemism, and conservation status.

## Sampling methods

### Study extent

**Figure 1.**
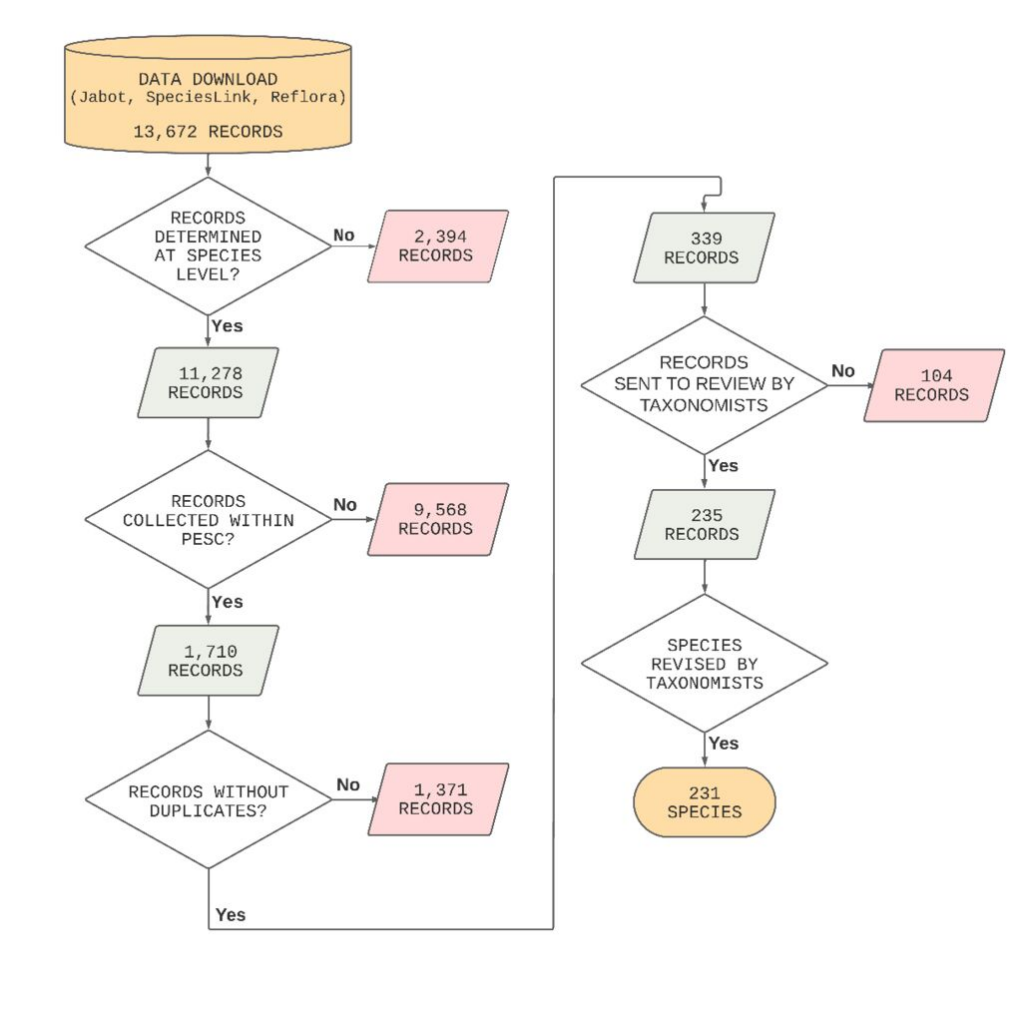
Stages of data cleaning to obtain a list of plants of "Parque Estadual da Serra da Concórdia", Rio de Janeiro, Brazil. The specimens kept on the list are shown in green, while the specimens removed are shown in red.

The list of all vascular plant specimens from the PESC was obtained through four searches in four online national databases: JABOT RB and JABOT Geral (JBRJ - Instituto de Pesquisas Jardim Botânico do Rio de Janeiro 2022), Herbário Virtual REFLORA (Reflora 2023), and INCT Herbário Virtual da Flora e dos Fungos, hereafter speciesLink (2022). An initial search was conducted using the filters "Concórdia" and "Santa Mariana" for "locality", which returned 234 unique records. Due to the limited number of initial records, the municipalities names ("Barra do Piraí" and "Valença") were included in the filters used. The following filters for location were applied: (1) "Barra do Piraí", (2) "Valença", (3) "Concórdia", and (4) "Santa Mariana". These searches resulted in a total of 13,672 records (JABOT RB = 1,291; JABOT Geral = 2,416; REFLORA = 3,348; and SpeciesLink = 6,617; Fig. 1).

The specimens identified to species were separated by manually, resulting in: JABOT RB = 998 (undetermined = 293), JABOT Geral = 1,925 (undetermined = 491), REFLORA = 2,790 (undetermined = 558), and speciesLink = 5,565 (undetermined = 1,052). Records with a locality outside the PESC area and another specific locality within (e.g. "Fazenda Santa Mônica", "Parque Natural Municipal do Açude da Concórdia", "Curral de Santa Mariana", "Cachoeira do Bonsucesso", and "Santuário de Vida Silvestre da Serra da Concórdia") were then removed from the list. After this process, the duplicates were removed, resulting in 339 records, which were filtered to retain one record for each species, prioritizing those with images in the databases. The raw data was subdivided into Microsoft Excel sheets, with determined (one record by name species), and undetermined records.

We used the online tool Plantminer species (Carvalho et al. 2010, Carvalho 2021) to align the species name with Flora e Funga do Brasil (2023). A preliminary list of 339 specimens was checked and validated by 24 taxonomists, using images available in herbaria and their online databases. The names adopted by the taxonomists were those accepted for publication of the final checklist of vascular plants of the PESC, was then published by Waga et al. (2023) in the Catálogo de Plantas das Unidades de Conservação do Brasil (2023).

The plots of species richness by genus and family for each plant group were made using the package "ggplot2" version 3.4.2 (Wickham 2016), developed for the R language. The geographic coverage data of the PESC were obtained through an analysis using the packages "raster" (Hijmans 2023), "sf" (Pebesma 2018), and "ggplot2" developed for R language (Wickham 2016). The altitudinal range data was obtained using the dataset name "alt", which is an aggregated data from SRTM 90m resolution (Jarvis et al. 2008, Hijmans 2023).

**Origin, endemism, and conservation status**: Information regarding the origin (native, naturalized, or cultivated) and endemism of species for the Brazilian Atlantic Forest and the state of Rio de Janeiro follows Flora e Funga do Brasil (2023), see also BFG (2018) and BFG (2021), and was obtained through Plantminer species (Carvalho et al. 2010, Carvalho 2021). The conservation status of species was obtained from the Official National Red List published by the Ministry of the Environment and Climate Change of Brazil (MMA, acronym in Portuguese from "Ministério do Meio Ambiente e Mudança do Clima"; MMA Ordinance No. 148/2022), through the database of Brazilian National Center for Plant Conservation (CNCFlora/JBRJ, acronym in Portuguese for "Centro Nacional de Conservação da Flora" of "Instituto de Pesquisas Jardim Botânico do Rio de Janeiro"), which serves as the IUCN SSC Brazil Plant Red List Authority (IUCN SSC BP-RLA).

## Geographic coverage

### Description

**Figure 2.**
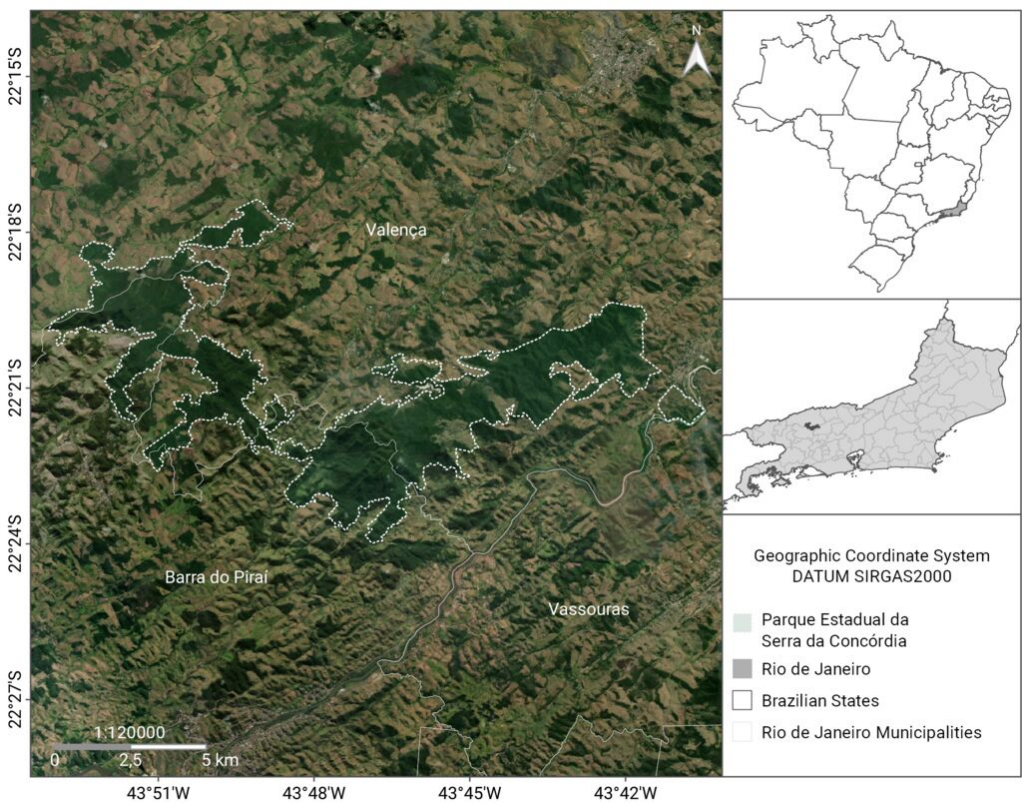
Location map of the “Parque Estadual da Serra da Concórdia”, Rio de Janeiro, Brazil.

The PESC covers the municipalities of Barra do Piraí and Valença, in the state of Rio de Janeiro, Brazil. When it was created in 2002, the area covered 804.41 ha. However, in 2016, the protected area was expanded and now consists of 5,952.11 ha (Fig. 2). The areas included in the list represent the entire region of "Fazenda Santa Mônica", "Açude da Concórdia" ("Parque Natural Municipal do Açude da Concórdia"; Fig. 3a), "Curral de Santa Mariana", "Cachoeira do Bonsucesso", and "Santuário de Vida Silvestre da Serra da Concórdia". The altitudinal gradient in the PESC varies between 343 and 1,007 m. The regional climate is classified as Cwa, which is a subtropical zone with dry winters and hot summers, and Cwb, a high-altitude subtropical zone with dry winters and temperate summers (Alvares et al. 2013). The annual mean rainfall from 1982 to 1990 was 1,285 mm (Spolidoro 2001). Despite the abandonment of the sites and the natural regeneration of some forest fragments, the region comprises an area of Semidecidous Seasonal Forest (Fig. 3b) remnants on the border of deforested areas and pastures (Fig. 3c, d).

### Coordinates

22°23'59"S and 22°17'22"S Latitude; 43°40'30"W and 43°53'07"W Longitude.

## Taxonomic coverage

### Description

The plant list for PESC contains a total of 231 species (Fig. 4) of 164 genera and 73 families, with 210 angiosperms (151 genera/62 families), 19 ferns (16 genera/13 families) and two lycophytes (*Selaginella* P.Beauv./Selaginellaceae). There are several reasons that could explain the low number of species recorded in PESC, such as fragmentation, deforestation, and the naturally lower richness found in Seasonal Forests compared to Rainforests. However, this low species count is also linked to the limited sampling and collection efforts in this area. There are very few botanical collections recorded in this region as a whole, which is reflected in the recorded richness for PESC. Additionally, only vouchers identified to species level, with images available online on data platforms, were considered for the species list of PESC. Nonetheless, the number of species is much higher than that found by Spolidoro (2001), who recorded only 85 species in their floristic survey in 0.3 ha.

The richest families of angiosperms in PESC are Fabaceae (41 species, 20%), Rubiaceae (15 spp., 7%), Asteraceae and Melastomataceae (10 spp., 5% each), and Bignoniaceae and Lauraceae (8 spp., 4% each; Fig. 5a); the richest families of ferns and lycophytes are Pteridaceae (5 spp., 24%), Polypodiaceae (4 spp., 20%), and Selaginellaceae (2 spp., 10%), the other families had one species each Fig. 5b. These families comprise 40% of angiosperms species and 54% of ferns and lycophytes species. A total of 35 families are represented only by one species (15%). The richest genera of angiosperms in PESC are: *Miconia* Ruiz & Pav. (7 spp.), *Inga* Mill. (5 spp.), and *Casearia* Jacq., and *Machaerium* Pers. (4 spp. each; Fig. 5c). Considering ferns and lycophytes, the richest genera are *Pteris* L. (5 spp.), *Selaginella* P.Beauv., and *Serpocaulon* A.R.Sm. (2 spp. each), while the other genera had one species each Fig. 5d. These genera comprise 13.2% of angiosperms and 56.2% of ferns and lycophytes, although a total of 127 genera are represented by only one species.

The richest angiosperm families registered in the PESC match partially the richest families in the Brazilian flora, except for Bignoniaceae and Lauraceae (BFG 2021). Among the richest genera, *Miconia* is also one of the genera with the highest number of species in the country (BFG 2021). Considering the “Paraíba do Sul” valley, Sales et al. (2018) studied five fragments between 10 and 211 ha (totaling 381 ha) and the total number of species from these areas was 301, with 29 species inventoried in the smallest fragment, and 177 in the largest. Fabaceae, Rubiaceae, and Lauraceae are among the richest families found in the Paraíba do Sul Valley (Sales et al. 2018), similarly to PESC.

## Traits coverage

There is only one non-native species from Brazil registered for the PESC: *Bidenssquarrosa* Kunth (Asteraceae, Flora e Funga do Brasil 2023), indicating a lack of collection efforts by botanists to document the occurrence of these plants. Sixty-three species from PESC are endemic to the Atlantic Forest (27%), being: Fabaceae (12 spp.), Melastomataceae, and Rubiaceae (4 spp. each) the richest families. Six species are endemic to the Atlantic Forest from Rio de Janeiro: *Lepidaploapersicifolia* (Desf.) H.Rob., *Adenocalymmacauliflorum* L.H.Fonseca & L.G.Lohmann, *Tovomitopsissaldanhae* Engl., *Swartziaglazioviana* Glaz. ex R.S.Cowan, *Besleriagrandifolia* Schott, and *Mollinedialowtheriana* Perkins.

Based on the Official National Red List (MMA Ordinance Nº 148/2022), eleven species are threatened with extinction: three are categorized as Endangered (*Dimorphandraexaltata* Schott, *Carinianalegalis* (Mart.) Kuntze, and *Zanthoxylumretusum* (Albuq.) P.G.Waterman), and eight are categorized as Vulnerable (*Xylopiabrasiliensis* Spreng., *Apuleialeiocarpa* (Vogel) J.F.Macbr., *Dalbergianigra* (Vell.) Allemão ex Benth., *Degueliahatschbachii* A.M.G.Azevedo, *Lepidaploapersicifolia* (Desf.) H.Rob., *Melanoxylonbrauna* Schott, *Swartziaglazioviana* (Taub.) Glaz., and *Cedrelaodorata* L.).

The species *Senegaliaparviceps* (Speg.) Seigler & Ebinger (Fabaceae) is the only species classified as Data Deficient (DD) and with restricted distribution, previously recorded only in the state of Paraná, Brazil (CNCFlora 2012, Martinelli and Moraes 2013). However, there is evidence of the presence of this species in other states in Flora e Funga do Brasil (2023) and an expansion of available data in the searched herbaria databases. The register for PESC is a new record for the flora from the state of Rio de Janeiro.

## Temporal coverage

### Notes

The floristic studies at the PESC began before its creation, with records from 1999 until 2018. The collection efforts dedicated to this area can be summarized into two main works: the first by Spolidoro (2001), with specimens recorded by M.L.C.V. Spolidoro and H.C. Lima, and the second effort conducted in the context of the project “Floristic inventory in state protected areas as a subsidy for the conservation of endemic and threatened with extinction species, and for forest restoration in the state of Rio de Janeiro”, led by CNCFlora/JBRJ and Secretariat for the Environment and Sustainability of Rio de Janeiro ("SEAS – Secretaria de Meio Ambiente e Sustentabilidade do Rio de Janeiro"), with specimens mainly recorded by C. Baez and M. Verdi.

## Usage licence

### Usage licence

Creative Commons Public Domain Waiver (CC-Zero)

## Data resources

### Data package title

A dataset of vascular plant species in Parque Estadual da Serra da Concordia, Rio de Janeiro, Brazil

### Resource link

https://doi.org/10.5281/zenodo.11044006

### Alternative identifiers

https://doi.org/10.15468/nkje8k; https://ipt.pensoft.net/resource?r=a-dataset-of-vascular-plant-species-in-parque-estadual-da-serra-da-concordia-rio-de-janeiro-brazil

### Number of data sets

1

### Data set 1.

#### Data set name

dataset_serra_concordia_state_park_v3.tsv

#### Data format

tsv, csv

#### Download URL

https://zenodo.org/records/11044006/files/dataset_serra_concordia_state_park_v3.tsv?download=1

#### Description

Dataset published by Deccache et al. (2024) containing information about the species of vascular plants from "Parque Estadual da Serra da Concórdia". It contains 231 species of vascular plants occurring in the Seasonal Semideciduous Forest and highlights endemic species of the Atlantic Forest and the IUCN risk of extinction categories according to CNCFlora/JBRJ. This data is also available on GBIF - Verdi (2024).

**Data set 1.** 

Column label	Column description
occurrenceID	A unique identifier code for each record.
collectionCode	Database where the specimen can be found.
institutionCode	Hebarium of origin of the cited specimen.
basisOfRecord	The specific nature of the data record.
catalogNumber	Specimen reference code in the herbarium.
phylum	The full scientific name of the division in which the taxon is classified.
family	The full scientific name of the family in which the taxon is classified.
scientificName	Full name of the taxon in accordance with the Flora e Funga do Brazil.
recordedBy	Main collector of the specimen.
recordNumber	Main collector number of the specimen.
country	Country where the the specimen was recorded.
countryCode	Code of the country where the specimen was recorded.
stateProvince	The name of the next smaller administrative region other than country (state, province, canton, department, region, etc.).
municipality	The full name of the next smaller administrative region other than county (city, municipality, etc.).
verbatimLocality	The original textual description of the place.
decimalLatitude	Latitude of the point of the specimen recorded.
decimalLongitude	Longitude of the point of the specimen recorded.
geodeticDatum	The ellipsoid, geodetic datum, or spatial reference system (SRS) upon which the geographic coordinates given in decimal Latitude and decimal Longitude is based.
establishmentMeans	Statement about whether a taxon has been introduced to a given place and time through the direct or indirect activity of modern humans.
endemism	Endemism of the species for the Mata Atlantica domain, based on the data of endemism and phytogeographic domain of the species obtained in Flora e Funga do Brazil.
conservationStatus	IUCN Red List category based on CNCFlora/JBRJ assessment.

## Additional information

The list of vascular plants from the "Parque Estadual da Serra da Concórdia" indicates that current inventories of flora in Brazil's protected areas contribute to improving the understanding of the gaps in Brazilian flora knowledge and supporting the proposal of efficient conservation actions. There were 63 species endemic to the Atlantic Forest and six endemic to the state of Rio de Janeiro, in addition to ten species classified in some threat category. Although sporadic expeditions have occurred in the "Parque Estadual da Serra da Concórdia", we emphasize the need to increase collection efforts in this protected area to expand occurrence records and plant diversity. Perhaps the low species richness recorded is a result of prolonged exploitation of the PESC area and its consequent edge effect. However, detailed studies should be conducted to verify this assumption.

## Figures and Tables

**Figure 3a.**
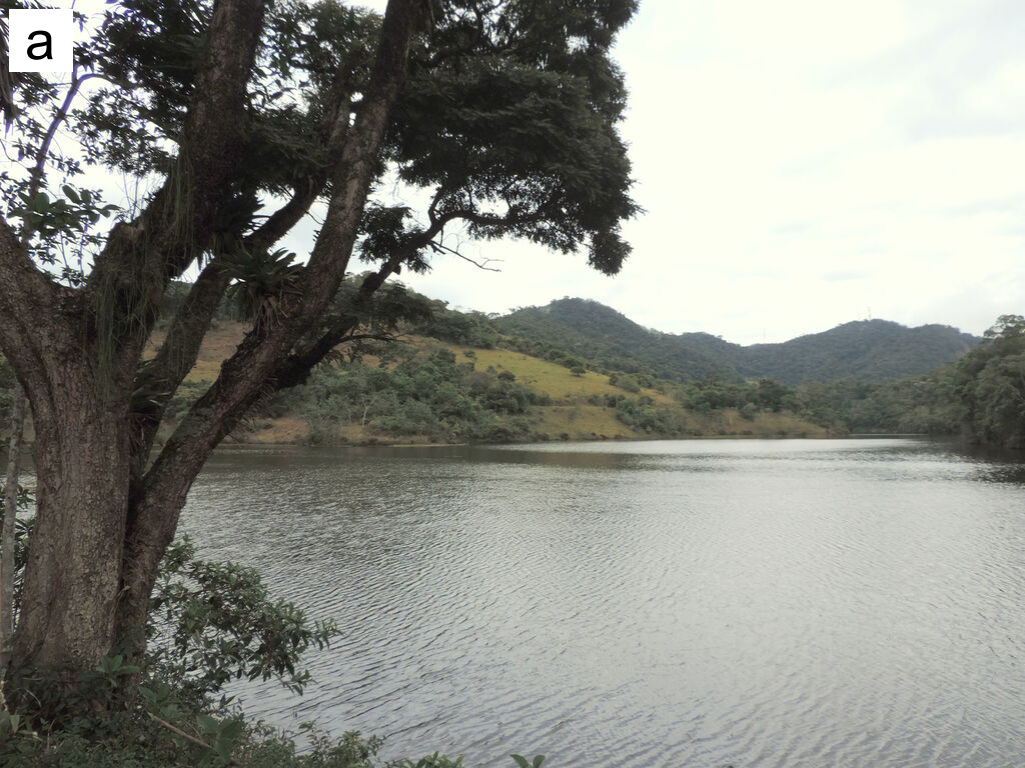
Parque Natural Municipal do Açude da Concórdia.

**Figure 3b.**
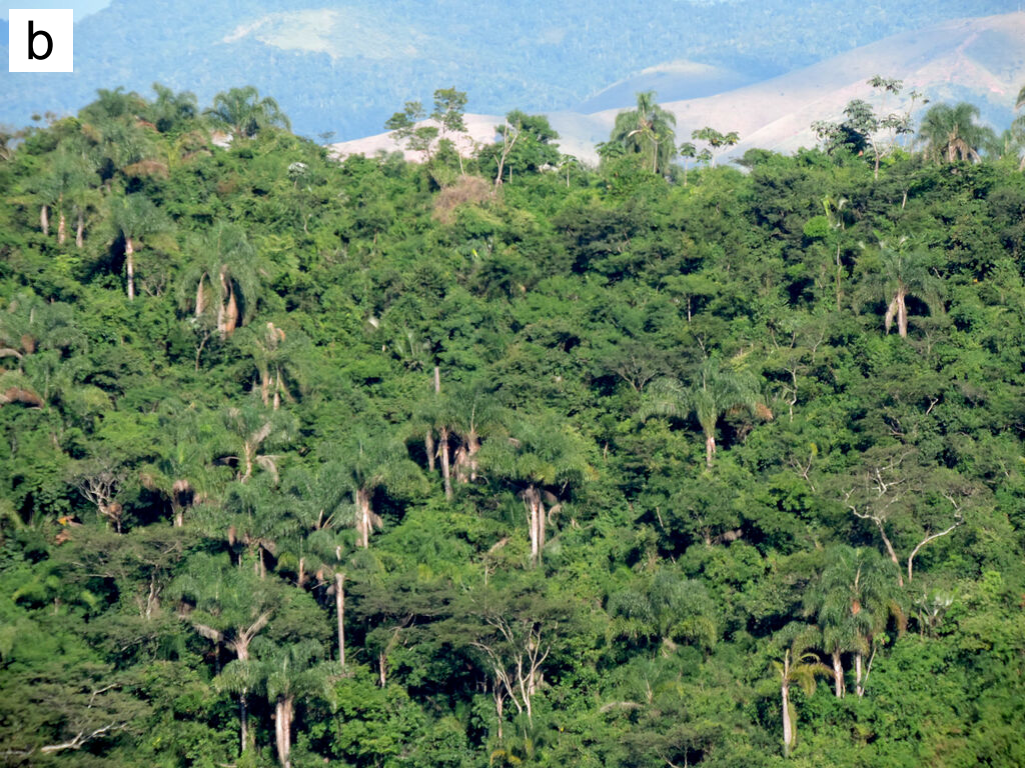
Scenery of the Semideciduous Seasonal Forest in the PESC.

**Figure 3c.**
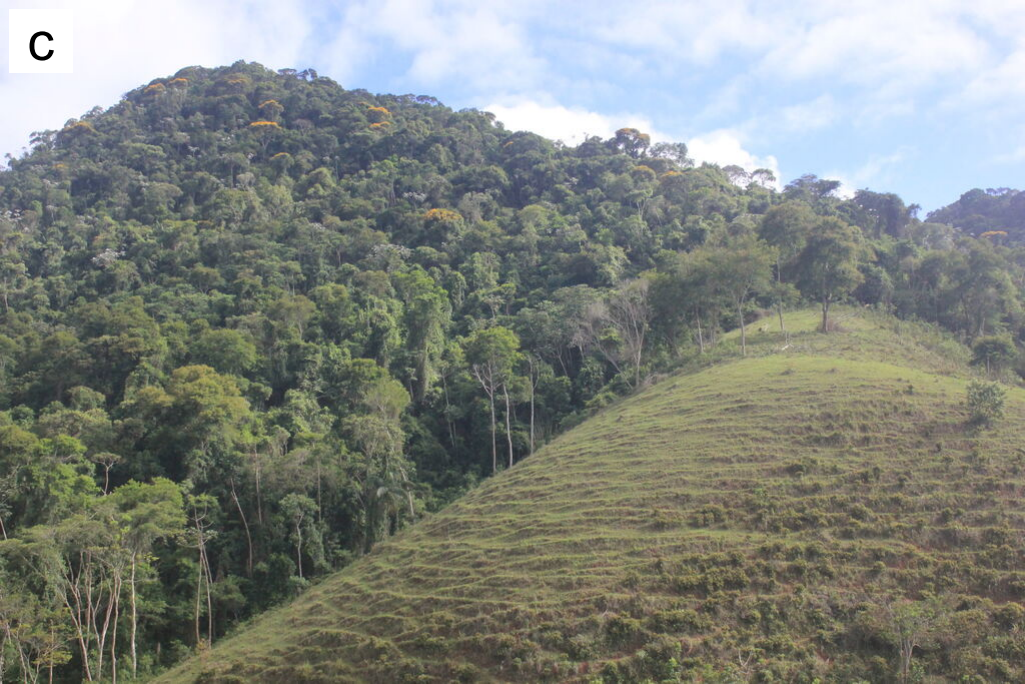
Deforested area on the border of the PESC.

**Figure 3d.**
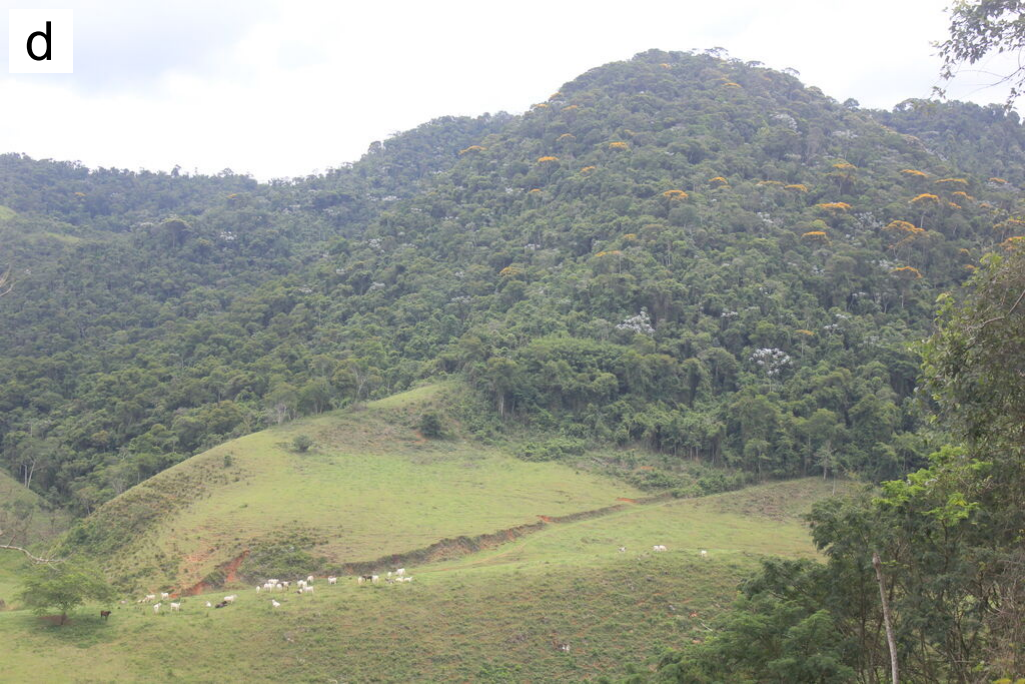
Pasture area, one of the anthropogenic pressures on the PESC.

**Figure 4a.**
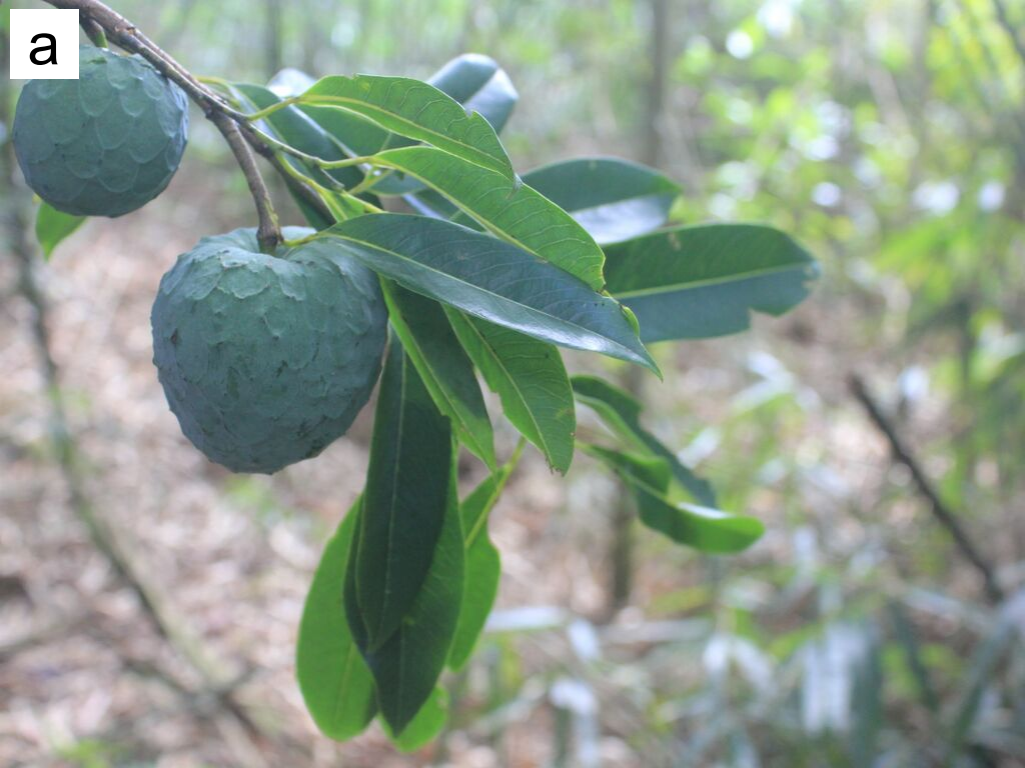
*Annonacacans* Warm. (Annonaceae).

**Figure 4b.**
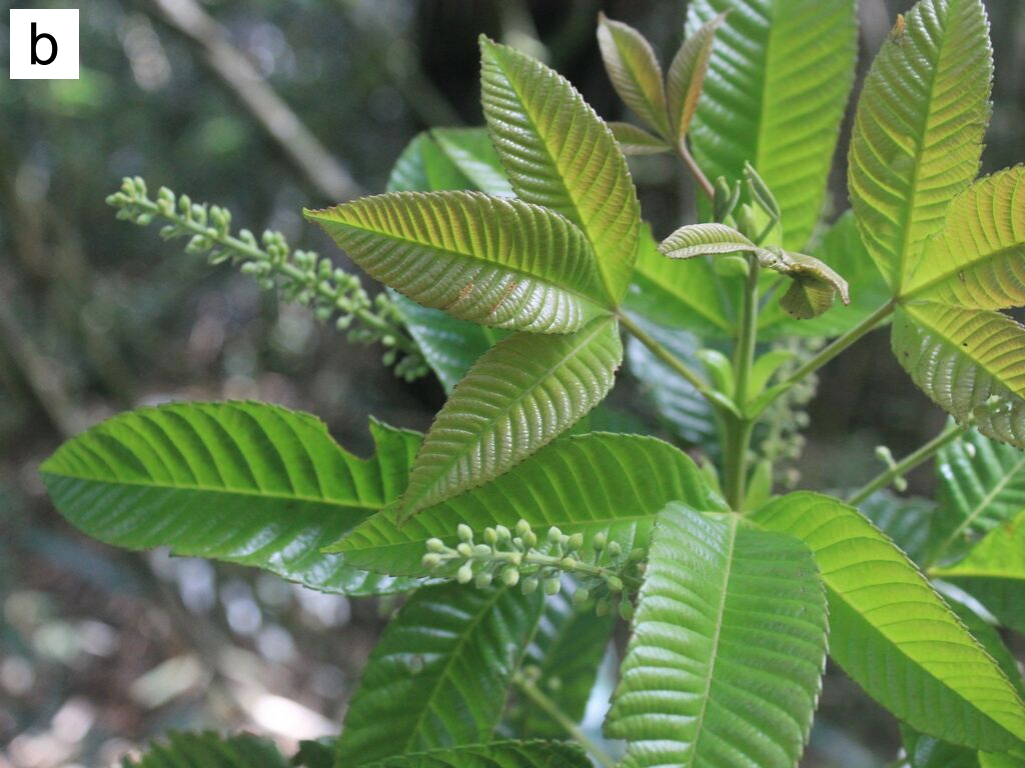
*Lamanoniaternata* Vell. (Cunoniaceae).

**Figure 4c.**
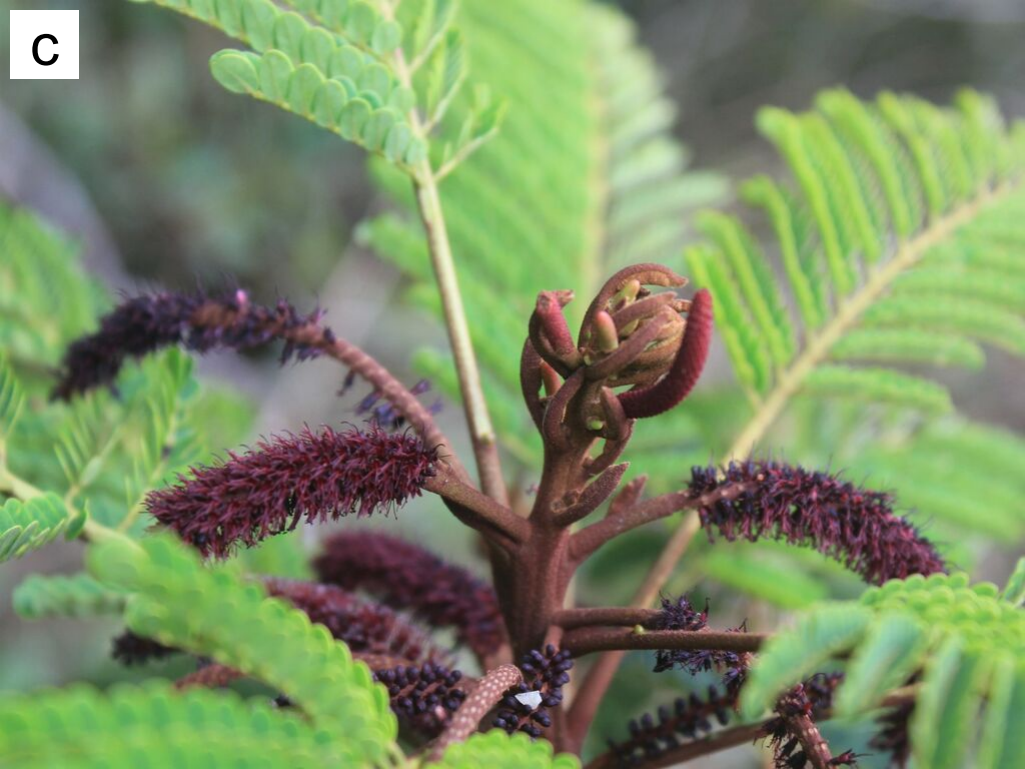
*Stryphnodendronpolyphyllum* Mart. (Fabaceae).

**Figure 4d.**
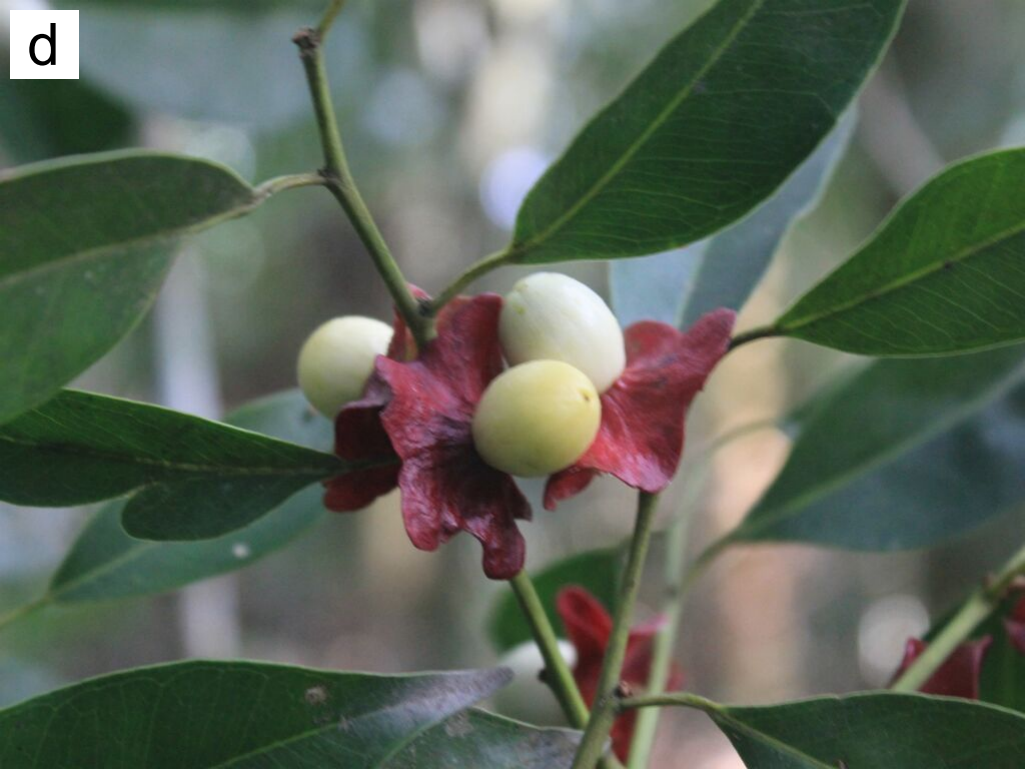
*Heisteriasilvianii* Schwacke (Erythropalaceae).

**Figure 4e.**
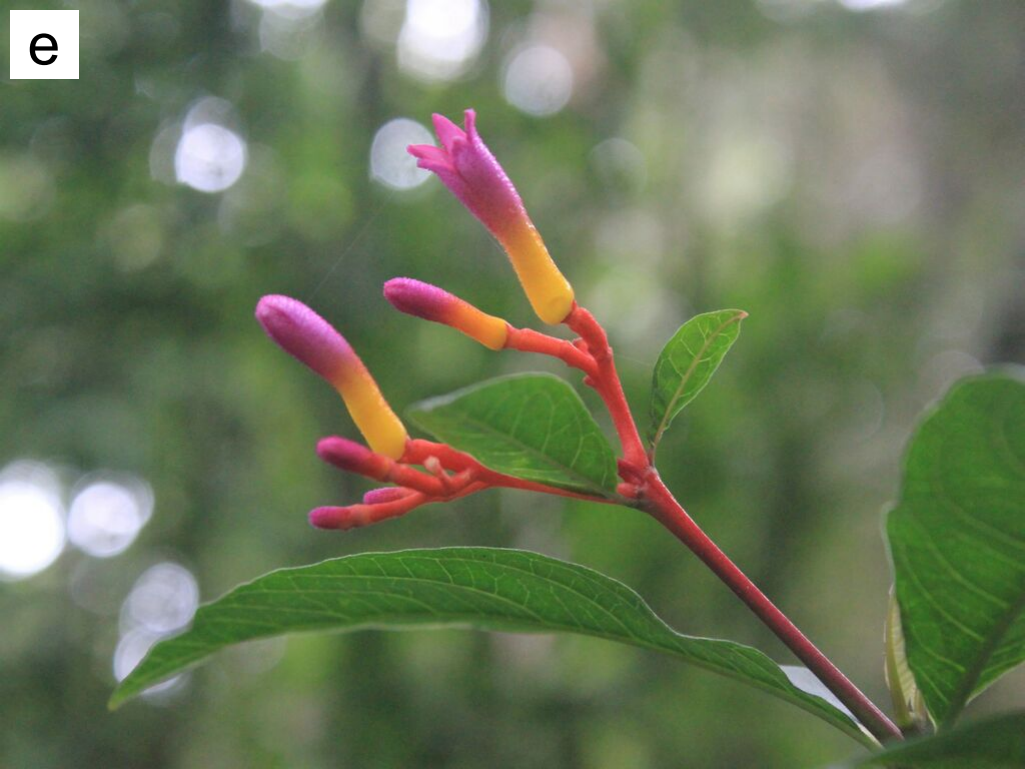
*Palicoureamarcgravii* A.St.-Hil. (Rubiaceae).

**Figure 4f.**
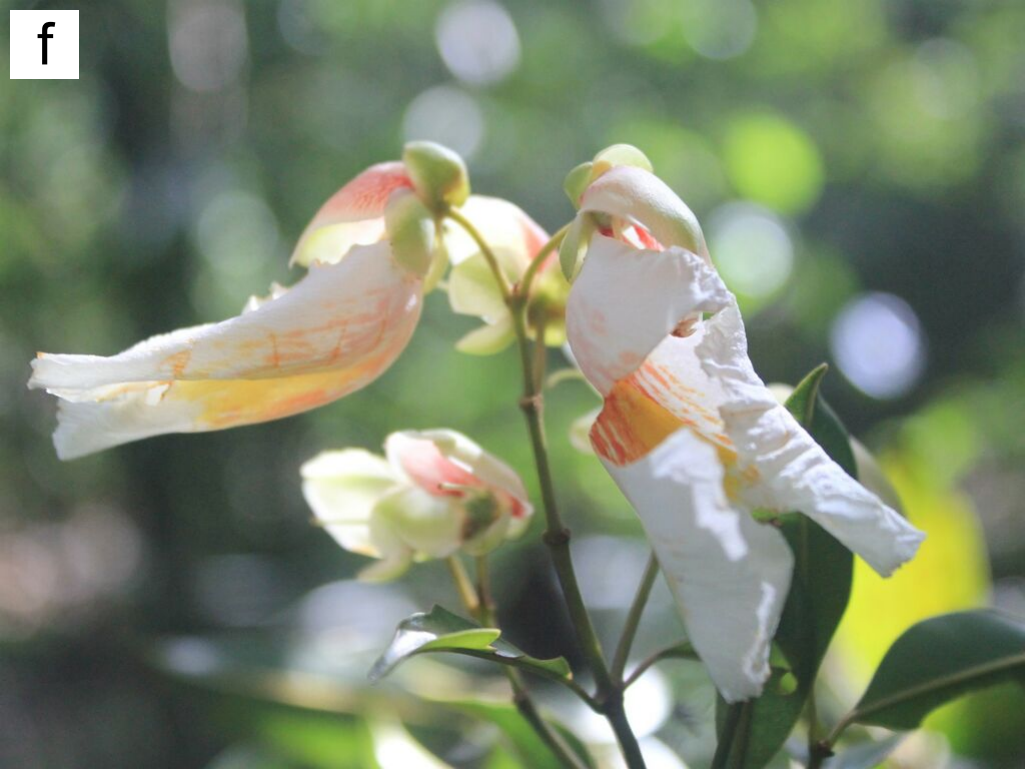
*Qualeagestasiana* A.St.-Hil. (Vochysiaceae).

**Figure 5a.**
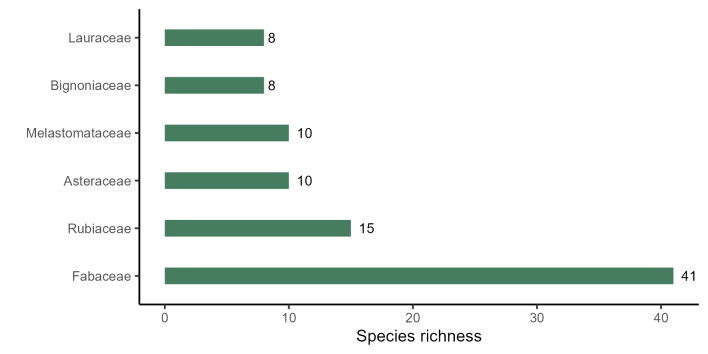
Richest families of angiosperms from PESC.

**Figure 5b.**
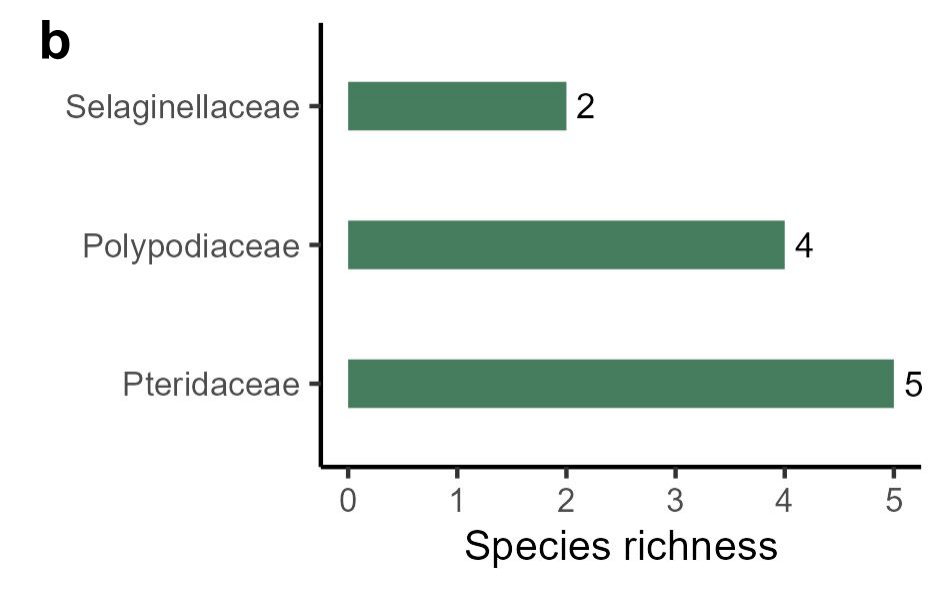
Richest families of ferns and lycophytes from PESC.

**Figure 5c.**
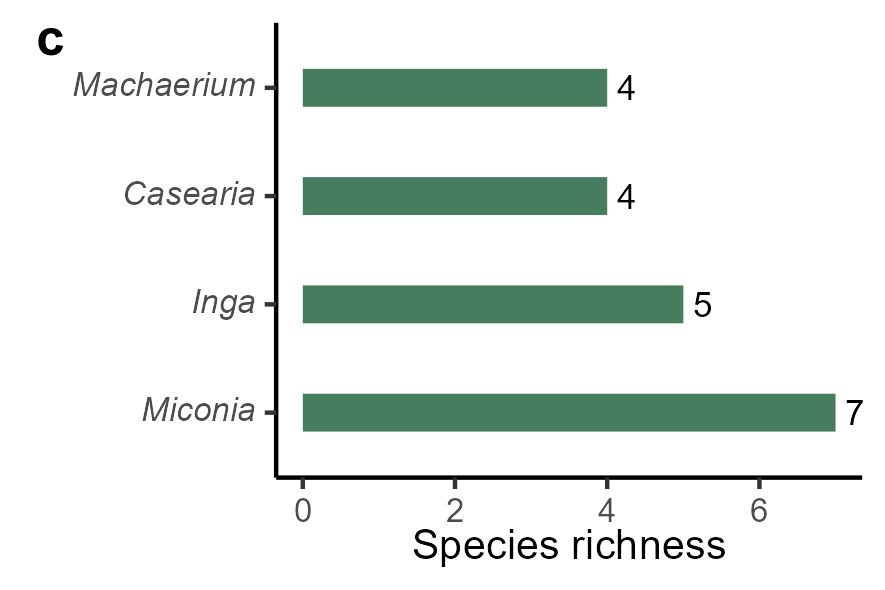
Richest genera of angiosperms from PESC.

**Figure 5d.**
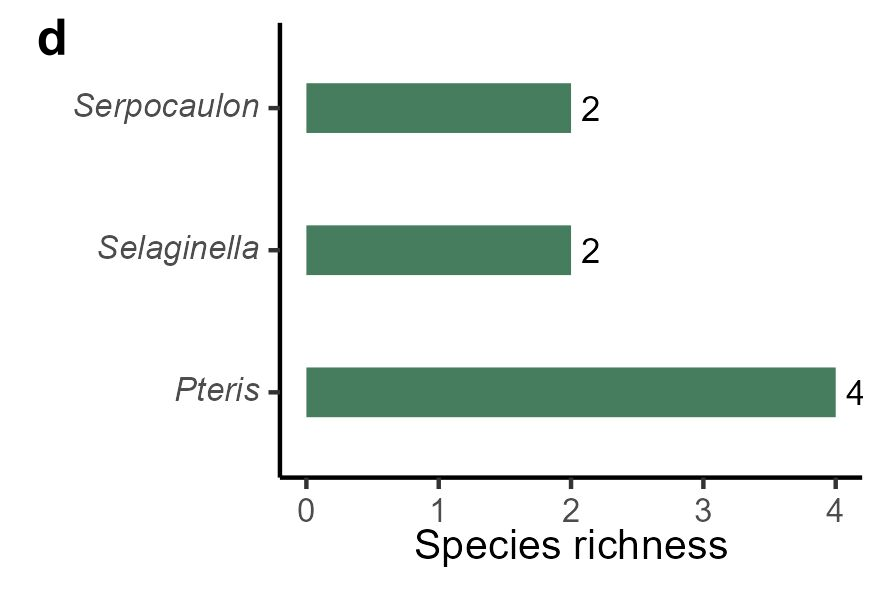
Richest genera of ferns and lycophytes from PESC.
